# Cardiac Rehabilitation Exercise in Korea

**DOI:** 10.1298/ptr.R0034

**Published:** 2025-07-19

**Authors:** Yun-cheol HA, Nan-soo KIM

**Affiliations:** 1 Irom Visiting Exercise Center, Republic of Korea; 2 Department of Physical Therapy, Catholic University of Pusan, Republic of Korea

**Keywords:** Cardiac rehabilitation, Exercise, South Korea

## Abstract

Objectives: This study aimed to systematically review exercise-based cardiac rehabilitation (CR) programs implemented in South Korea since their inclusion in the National Health Insurance System in 2017. Methods: A systematic literature search was conducted using international and Korean databases for studies published after January 2017. The inclusion criterion was exercise-based CR intervention in patients with cardiac disease in South Korea. The methodological quality of each study was assessed using the Physiotherapy Evidence Database scale. Results: A total of 11 studies met our inclusion criteria. Most CR programs are hospital-based, whereas some incorporate home-based, aquatic, or forest environments. The exercise prescriptions followed the FITT (frequency, intensity, time, and type) principle and often included aerobic and high-intensity interval training. The reported outcomes included improvements in cardiopulmonary fitness, physical activity, and psychological well-being. Despite their clinical effectiveness, participation in CR programs remains low in Korea owing to accessibility and institutional limitations. Conclusions: Exercise-based CR programs in Korea demonstrated clinical benefits and diversified delivery models. Further efforts are required to enhance accessibility and promote wider adoption through policy and infrastructure development.

## Introduction

Cardiac rehabilitation (CR) is defined as a multidisciplinary intervention aimed at enhancing cardiovascular health and reducing the risk of recurrence in patients with cardiovascular disease (CVD)^1)^. The World Health Organization (WHO) defines CR as “the sum of activities required to ensure patients the best possible physical, mental, and social conditions, so that they may, by their own efforts, resume and maintain optimal functioning in their community”^2)^. This comprehensive approach, which includes exercise therapy, risk factor management, nutritional counseling, psychosocial support, and patient education, is essential for secondary prevention of CVDs^3)^.

The American Heart Association (AHA) and the American Association for Cardiovascular and Pulmonary Rehabilitation (AACVPR) emphasize that an effective CR program should include exercise therapy prescribed by a physician, modification of cardiovascular risk factors, psychosocial assessment, and assessment of patient prognosis^4)^. The goal of such an intervention program is to improve the patients’ quality of life, restore physical function, and prevent the progression of CVD.

The effectiveness of CR has been demonstrated in several studies, with participants in CR programs reporting a 25% reduction in cardiovascular mortality and an 18% reduction in hospitalization rates^5)^. Furthermore, systematic CR has been shown to enhance maximum oxygen intake (VO_2_max) by an average of 15%–20%, which has been linked to a 25% reduction in mortality for every 1-unit increase in the metabolic equivalent of task, indicating cardiopulmonary endurance^6)^. These functional improvements facilitate the patients’ return to daily life and contribute to improved overall health.

In South Korea, CVD accounts for the 2nd highest proportion of deaths, with 64.8 deaths per 100000 people^7)^. However, despite the strong clinical evidence for CR, the implementation of CR programs in South Korea remains insufficient. Of the 164 hospitals in Korea that perform percutaneous coronary intervention, only 47 (28.7%) operated a CR program; of these, 8 lacked a systematic program and the remaining 39 performed limited activities^5)^. According to data from the Health Insurance Review and Assessment Service, the number of registered cardiovascular patients in Korea as of 2023 is approximately 1.9 million, of whom only 15646 received CR treatment^8)^. The number of prescriptions for CR increased from 4443 in 2017 to 15646 in 2023, representing a 252.1% increase over 7 years^8)^. However, the usage rate remains lower than that observed in developed countries.

Physical activity (PA) plays a pivotal role in CR, and exercise has a positive effect on various cardiovascular risk factors, including high blood pressure, high-density lipoprotein levels, body weight, and insulin sensitivity^9)^. This contributes to the prevention of CVDs and reduces mortality. Consequently, a multidisciplinary approach is imperative for CR, and exercise remains a pivotal component^1)^. Implementation of a safe and gradual exercise program is crucial for enhancing patient prognosis. However, significant variations exist among studies concerning exercise type, frequency, intensity, and duration, resulting in a paucity of consistent guidelines^10)^.

Considering the identified limitations, it is imperative to formulate exercise prescription guidelines that are congruent with the Korean healthcare environment. This study aimed to systematically analyze recent trends and practical applications of exercise prescriptions for CR in patients with cardiac disease in South Korea since their inclusion under the National Health Insurance System in 2017. This study seeks to contribute to the revitalization and expansion of CR in Korea by presenting evidence-based exercise prescription guidelines optimized for the Korean healthcare environment.

## Methods

A literature search was conducted from January 1 to February 8, 2025, using the following databases and platforms: Cochrane, Embase, PubMed, Scopus, DBpia, KISS, RISS, KCI, SCIENCE_ON, National Assembly Electronic Library, and Google Scholar. The search terms used were heart disease, cardiac rehabilitation, South Korea, and Republic of Korea. These terms were combined using the Boolean operators OR and AND to include controlled words and natural languages. To supplement the electronic search, a manual search was also conducted using keywords.

The selection criteria for this study were domestic (South Korean) studies that reported appropriate studies on CR programs for patients with cardiac disease that met the core questions of the PICO (Population: heart disease AND South Korea, Intervention: cardiac rehabilitation, Compare: none, Outcome: frequency, intensity, time type, etc.). Studies that were covered by the National Health Insurance System after January 2017 were selected. The exclusion criteria included studies deemed irrelevant to PICO, studies for which the original text could not be verified, studies that were currently ongoing, and studies for which appropriate results had not been reported.

During the selection process for the relevant literature, a risk of bias assessment for each study was conducted independently by 2 authors using the Physiotherapy Evidence Database (PEDro) scale. The PEDro scale consists of 10 items rated as “yes” or “no.” Studies with a total score of 6 or more points were selected as high-quality studies. The selection process involved a multifaceted evaluation of the studies, encompassing various methodological aspects such as random allocation, allocation concealment, baseline equivalence, blinded treatment, blinded subjects, blinded assessors, analysis of intention-to-treat, adequacy of follow-up, between-group statistical analysis, and point-estimate variability. Any discrepancies between the reviewers were resolved through consensus.

## Results

**Fig. 1.**
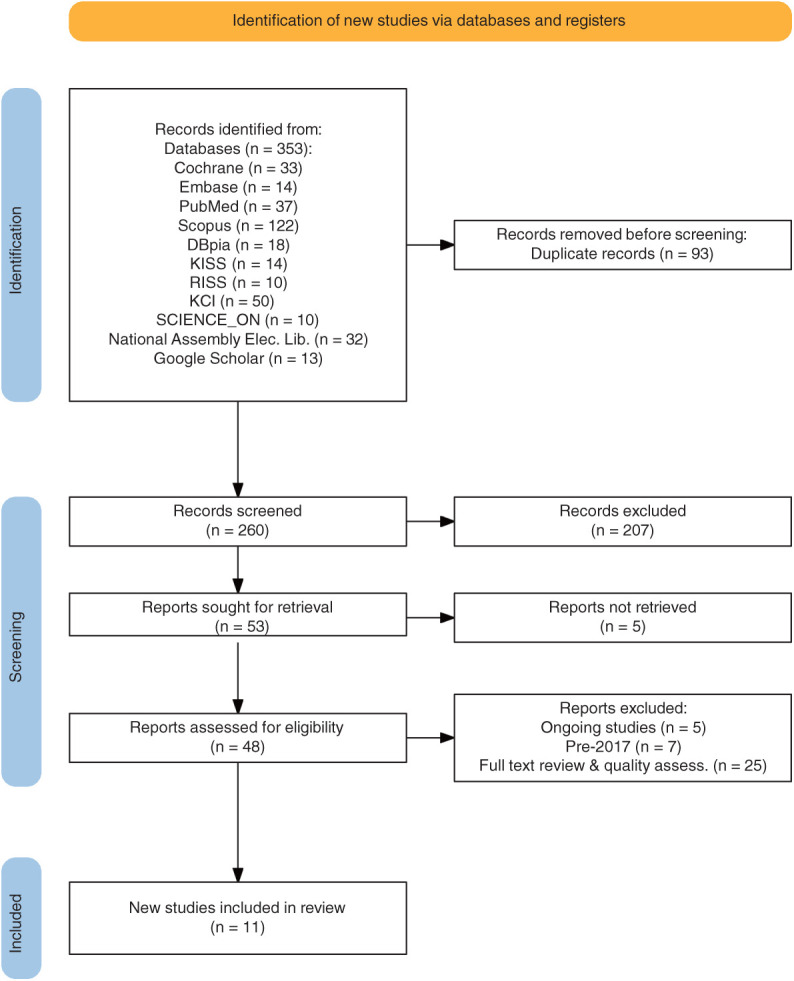
PRISMA flow diagram.

The literature search process for this study is demonstrated in the Preferred Reporting Items for Systematic Reviews flow diagram, and the final 11 studies^11–21)^ were incorporated into the literature review (Fig. 1). A qualitative evaluation of the literature revealed that the final 11 studies^11–21)^ adequately described the current state of exercise-based CR in Korea. The purpose, design, method, and random allocation of subjects were described in accordance with the selection and exclusion criteria. Four studies^14,17,19,20)^ were concealed regarding the allocation of study subjects, 11 studies^11–21)^ had similar or identical baseline characteristics, 1 study^17)^ had a masked study subject, no studies had a masked physiotherapist, 2 studies^19,20)^ had a masked researcher, and 8 studies^11–13,15–18,21)^ included at least 85% of the participants in the final analysis. All 11 studies^11–21)^ adhered to the intended protocol, provided statistical comparisons between the groups, and reported specific numerical outcome values. Their PEDro scores were 6 out of 10 in 8 studies^11–16,18,21)^, 7 in 2 studies^19,20)^, and 8 in 1 study^17)^ (Table 1) .

**Table 1.** Literature quality assessment (PEDro scale)

Study	Eligibility criteria	Random allocation	Concealed allocation	Baseline similarity	Blinding (subjects)	Blinding (therapists)	Blinding (assessors)	Follow-up >85%	Intention-to-treat analysis	Comparison between groups	Point estimates and variability	Quality score (0–10)
Kim et al. (2019)^11)^	Y	Y	N	Y	N	N	N	Y	Y	Y	Y	6
Jung et al. (2024)^12)^	Y	Y	N	Y	N	N	N	Y	Y	Y	Y	6
Lee et al. (2024)^13)^	Y	Y	N	Y	N	N	N	Y	Y	Y	Y	6
Lee (2022)^14)^	Y	Y	Y	Y	N	N	N	N	Y	Y	Y	6
Ryu, et al. (2018)^15)^	Y	Y	N	Y	N	N	N	Y	Y	Y	Y	6
Lee et al. (2017)^16)^	Y	Y	N	Y	N	N	N	Y	Y	Y	Y	6
Nam et al. (2024)^17)^	Y	Y	Y	Y	Y	N	N	Y	Y	Y	Y	8
Ahn et al. (2021)^18)^	Y	Y	N	Y	N	N	N	Y	Y	Y	Y	6
Kim et al. (2020)^19)^	Y	Y	Y	Y	N	N	Y	N	Y	Y	Y	7
Jo et al. (2024)^20)^	Y	Y	Y	Y	N	N	Y	N	Y	Y	Y	7
Choi et al. (2018)^21)^	Y	Y	N	Y	N	N	N	Y	Y	Y	Y	6

PEDro, Physiotherapy Evidence Database; N, no; Y, yes

The exercise-based CR programs implemented in the 11 selected studies^11–21)^ are outlined in Table 2. The total number of subjects in these studies was 444, all of whom were enrolled in Korea. Most CR studies have been hospital-based, with a small number of home-based studies also conducted. Studies have also been conducted using forests and swimming pools as alternative settings. The study participants included patients with various cardiovascular conditions, such as acute myocardial infarction, atrial fibrillation, coronary artery disease, coronary artery bypass grafting, and pacemaker implantation. The CR phase was predominantly Phases II and III, with the objective of achieving functional recovery, and Phase I was incorporated into a number of studies.

**Table 2.** The reality of cardiac rehabilitation exercise in Korea

Study	CR setting	DC	Patients	CR phase	CR monitoring	Ex. freq.	Exercise intensity	Exercise time	Exercise type	Program	Total dur.	Other education	CR-A (phys.)	CR-A (other)
Kim et al. (2019)^11)^	Hospital	A-fib	EG = 13	None	None	Twice 1-wk apart	10–70 W	15 min	RBE (body tilt 30°)	GI (↑15 W/2min)	1 wk	None	HRV, CO, RPP	None
			CG = 13						UBE					
Jung et al. (2024)^12)^	Hospital, home	AMI, HF	EG = 7	II	None	2/wk	Mod-Vig Int. & HRR 40%–80%	150 min	AE & BWRE	None	6 wk	MBCP & NC	None	HRQoL, Dep, Anx, Type D, Stress
			CG = 6									SPE		
Lee et al. (2024^13)^	Urban forest, home	PCI	EG = 17	None	MDT	3/wk	Mod-Vig Int. & HRR 40%–80%	150 min	AE & BWRE, UE-UF	None	12 wk	ILE	CRE	BC, BG, HV, TC
		HR-CAD	CG = 11											
Lee (2022)^14)^	Hospital, home	CABG	EG = 16	I, II	None	3/wk	RHR+20	30–40 min, 2/day	LLE	None	4 wk	None	CPET, PFT, HST	None
			CG = 18				RPE 11–13							
Ryu et al. (2018)^15)^	Home	CHF	EG = 28	III, IV	Patient	None	Mod-Vig Int.	None	PA	None	1 year	None	CPET	Readmission
			CG = 19				NHE		No exercise					
Lee et al. (2017)^16)^	Hospital, swimming pool	CAD, OA	EG = 20	II, III	CR specialist	3/wk	HRR 50%–65%	30 min	Aqua walking	None	24 wk	None	CPET, BAI	Blood test, BDI, BAI, WHO-QOL
			CG = 21				HRR 50%–65% (adj. −15 to 17 bpm)		Treadmill/track walking					
			CG = 19	None	None	None	None	None	None	None				
Nam et al. (2024)^17)^	Hospital	AMI	EG = 30	II, III	MD, PT	2/wk	VO_2_max 95%–100%	50 min	Treadmill	2 wk Adapt -> 7 wk Progressive	9 wk	None	1st Outcome (CPET, MacNew HRQoL)	2nd Outcome (6MWT, Others)
			EG = 29				VO_2_max 85%							
			CG = 32				RPE 11–13							
Ahn et al. (2021)^18)^	Hospital	PPI	EG = 12	II	MDT	1–3/wk	HRR 55%–70%	60 min	Flexibility, aerobic, resistance exercise	None	4 wk	Individualized education	6MWT, CPET, Muscle Strength	SF-36
			CG = 15	None	None	None	None	None	None					
Kim et al. (2020)^19)^	Hospital	ACS	EG = 23	None	MD, PT	3/wk	HRR 95%–100%	45 min	Treadmill walking	2 wk Adapt -> 4 wk Progressive	6 wk	None	CPET	Blood test, ECHO
			CG = 24				HRR 85%	50 min						
Jo et al. (2024)^20)^	Mobile, home	AMI	EG = 24	II, III	MD, PT	3/wk	RPE 11–13 and prescribed intensity	60 min	Walk, run, cycle, resistance exercise	↑/2 wk	6 wk	ILE	CPET, KASI	EQ-5D-5L, PHQ-9
			CG = 24											
Choi et al. (2018)^21)^	Hospital	MI	EG = 23	II	None	1–2/wk	HRmax 85%–100%	48 min	Treadmill	None	9–10 wk	None	CPET, 6MWT, KASI	HADS, PHQ-9, ISI, FSS
			CG = 21				HRmax 60%–70%							

CR, cardiac rehabilitation; DC, disease condition; Ex.freq., exercise frequency; Total dur., total duration; CR-A, cardiac rehabilitation assessment; phys, physical test; A-fib, atrial fibrillation; EG, experimental group; CG, control group; wk, week; W, Watts; RBE, recumbent bicycle ergometer; UBE, upright bicycle ergometer; GI, gradual increase; HRV, heart rate variability; CO, cardiac output; RPP, rate pressure product; AMI, acute myocardial infarction; HF, heart failure; Mod-Vig Int., moderate-to-vigorous Intensity; HRR, heart rate reserve; AE & BWRE, aerobic exercise and body weight resistance exercise; MBCP & NC, mindfulness-based counseling program and nutrition counseling; SPE, secondary prevention education; HRQoL, health-related quality of life; Dep, depression; Anx, anxiety; Type D, Type D personality; PCI, percutaneous coronary intervention; HR-CAD, high-risk coronary artery disease; MDT, multidisciplinary team; UE-UF, unstructured exercise in an urban forest; ILE, intensive lifestyle education; CRE, cardiorespiratory endurance; BC, body composition; BG, blood glucose; HV, hemodynamic variables; TC, total cholesterol; CABG, coronary artery bypass graft; RHR, resting heart rate; RPE, rating of perceived exertion; LLE, lower limb ergometer; CPET, cardiopulmonary exercise testing; PFT, pulmonary function test; HST, handgrip strength test; CHF, chronic heart failure; NHE, no home based exercise; PA, physical activity; CAD, coronary artery disease; OA, osteoarthritis; BAI, Beck Anxiety Inventory; BDI, Beck Depression Inventory; WHO-QOL, World Health Organization Quality of Life questionnaire; MD, medical doctor; PT, physical theraphyist; MacNew HRQoL, MacNew Heart Disease Health-related Quality of Life scores; 6MWT, 6-minute walk test; PPI, permanent pacemaker implantation; SF-36, 36-Item Short Form Health Survey; ACS, acute coronary syndrome; ECHO, echocardiography; KASI, Korean Activity Scale/Index; EQ-5D-5L, EuroQol 5-Dimension 5-Level questionnaire; MI, myocardial infarction; PHQ-9, Patient Health Questionnaire-9; HADS, Hospital Anxiety and Depression Scale; ISI, Insomnia Severity Index; FSS, Fatigue Severity Scale

The exercise-based CR program was based on the FITT (frequency, intensity, time, and type) standards established by the American College of Sports Medicine (ACSM)^22)^. The frequency of exercise (F) varied across studies, but recommendations generally suggest 3 or more sessions per week as optimal, with 1–3 sessions per week being the most prevalent. Exercise intensity (I) was categorized as moderate to high, with intensity indicators including heart rate reserve (HRR), VO_2_max, and rating of perceived exertion (RPE). A significant proportion of the included studies employed high-intensity interval training (HIIT), with the exercise time (T) ranging from 15 to 60 min, including warm-up and cool-down periods, though most studies spanned between 30 and 50 min. The exercise type (T) primarily comprised general aerobic activities such as treadmill walking, cycling, and water walking, with some studies also incorporating resistance exercise utilizing the patient’s own body weight. The exercise program was meticulously designed to ensure patient safety by gradually increasing exercise intensity. In some studies, the frequency, intensity, and duration of exercise were adjusted systematically each week. In addition, Mindfulness-Based Counseling Program (MBCP), nutrition counseling, and education on lifestyle improvement and risk factor management were provided to ensure management and continuity.

Pre- and post-program assessments were conducted to assess the cardiopulmonary exercise testing (CPET) and the 6-minute walk test (6MWT) scores. The psychological status of the participants was assessed using standardized instruments, including the 36-Item Short Form Health Survey (SF-36), EuroQol 5-Dimension 5-Level Questionnaire (EQ-5D-5L), Hospital Anxiety and Depression Scale (HADS), Patient Health Questionnaire-9 (PHQ-9), and Fatigue Severity Scale (FSS) to evaluate quality of life, depression, anxiety, and fatigue, respectively. The findings of these studies indicate the safety of exercise-based CR and highlight the evidence supporting its necessity. In particular, studies that applied HIIT showed that it was more effective than conventional moderate-intensity continuous training (MICT) in enhancing cardiopulmonary function, PA, and psychological well-being^17,19,21)^.

Recent studies have demonstrated the efficacy of novel approaches to CR, including home-based rehabilitation utilizing mobile applications^20)^ and CR in urban forests^13)^. These findings suggest the potential for diversification and enhanced accessibility of CR programs in the future.

The results of the present study demonstrated the safety and effectiveness of Korean CR programs in improving cardiovascular function, PA, and psychosocial well-being. In particular, the results suggest that methods such as HIIT may be more effective than the existing moderate-intensity methods.

## Discussion

We systematically reviewed 11 studies on CR published since 2017, when it became a medical care benefit in South Korea, aiming to analyze the types, effects, and applications of exercise prescriptions. The analysis revealed that most domestic CR programs were hospital-based. However, recent initiatives have aimed to address patients’ diverse needs by using multidisciplinary approaches and incorporating interventions in various environments, such as forests and swimming pools, as well as digital health technology and psychotherapy.

Exercise prescription in CR in the analyzed studies was based on the FITT principles of the ACSM^22)^. The exercise types were predominantly aerobic, with a frequency of at least 3 times per week being the most common, and the intensity was moderate-to-high (HRR 40%–80%) based on objective indicators such as HRR, VO_2_max, and RPE. In the 2nd phase of CR, the conventional MICT and HIIT were compared. The results showed significant improvements in cardiovascular function, activity state, depression, and fatigue in the HIIT group compared with those in the MICT group^21)^. Recent studies have indicated that the maximal-intensity aerobic interval training (MAIT) group, which performed aerobic exercise at 95%–100% of HRR for CR patients, demonstrated superior improvements in VO_2_max compared with the high-intensity aerobic interval training (HAIT) group, which performed at 85% of HRR^17,19)^. The MAIT group also exhibited higher safety levels. This finding further supports the notion that high-intensity exercise can be safely performed under appropriate evaluation and monitoring conditions, as emphasized in recent guidelines from the AHA and AACVPR^4)^. Consequently, high-intensity exercises such as HIIT may be a viable and effective strategy for patients in Korea, except for those at high risk.

The environment in which CR is conducted changes from hospital-based to community-based depending on the rehabilitation stage. Recently, the rehabilitation environment and lifestyle have also been studied as factors that affect the effectiveness of interventions. A CR program conducted in an urban forest environment demonstrated improvements in body composition, blood sugar levels, hemodynamic variables, total cholesterol levels, and cardiorespiratory endurance comparable to those of a traditional CR program, without any safety-related concerns^13)^. In a separate study, aquatic walking was utilized as a CR modality for elderly patients with coronary artery disease and lower limb arthritis, yielding outcomes comparable to those observed in treadmill walking^16)^. Consequently, it was proposed that aquatic exercise could serve as a substitute modality for elderly patients requiring CR, as it mitigates joint strain. Access to a safe space for exercise at home or in the community has been identified as a core component of CR programs^4)^. The extant literature indicates that CR programs tailored to patient characteristics, such as age and environment, can help patients adopt healthy lifestyles and sustain their participation in CR programs.

Home-based CR has been demonstrated to be a significant factor in reducing rehospitalization rates^15)^. A home-based CR exercise program utilizing a mobile application has been shown to be as effective as conventional home-based CR with verbal supervision via telephone for enhancing exercise capacity (VO_2_max)^20)^. The use of mobile applications in CR has proven effective in promoting the maintenance of exercise and enhancing self-management. This finding highlights the potential of mobile apps as a pragmatic alternative for patients grappling with accessibility and temporal and financial constraints that often hinder their participation in hospital-based CR programs.

Psychological problems have been shown to increase the risk of CVD and reduce CR completion^4)^. Therefore, a comprehensive approach that considers psychosocial issues is required for CR. However, CR programs in Korea lack comprehensiveness and are not staffed with enough professionals from multidisciplinary teams to implement them effectively^23)^. Conversely, comprehensive CR incorporating an MBCP has been demonstrated to enhance anxiety and physical and mental health-related quality of life, while concurrently increasing participants' satisfaction with and engagement in the program^12)^. These findings imply that a multidisciplinary strategy that incorporates psychological factors can have a favorable influence on patient rehabilitation persistence and overall health behavior enhancement.

The number of CR treatments in Korea has increased from 2017 to 2023; however, the participation rate is only 0.9% of the total number of patients with cardiac disease^8)^. This is significantly lower than the 20%–30% participation rate in CR in the United States^24)^ and far behind international standards. The primary factors contributing to this suboptimal participation rate include the lack of adequate CR infrastructure, limited access to rehabilitation institutions, and the high financial costs of treatment and participation^25)^. This poses significant challenges, particularly for older adults, low-income populations, and residents of rural and coastal areas. Considering these challenges, recent initiatives have sought to enhance accessibility, increase adherence (or completion rate), and reduce costs by transitioning from conventional hospital-based models to home-based CR programs. This transition incorporates various community infrastructures and digital health technologies, such as mobile applications. However, the institutionalization of such comprehensive CR programs necessitates the enhancement of insurance coverage and institutional support, promotion of awareness among medical personnel and policymakers, reinforcement of education for related occupations, and revitalization of multidisciplinary rehabilitation, including psychological counseling and nutritional education^23)^.

This study systematically reviewed the domestic literature on exercise-based CR but included only domestic studies published since 2017, thus excluding previous studies and foreign research findings. Most of the included studies were limited, short-term studies conducted at a single institution, resulting in a small sample size and a lack of diversity in the patient population. Furthermore, the studies that addressed exercise prescriptions and effects used a variety of evaluation tools and intervention methods, which made quantitative comparisons through meta-analysis difficult. Consequently, future multicenter, long-term follow-up studies and strategic meta-analyses are necessary.

## Conclusions

This study systematically reviewed the actual application and clinical effects of exercise prescriptions, focusing on the CR programs implemented in Korea. The results demonstrate that CR in Korea is mainly based on a traditional hospital-centered approach based on the FITT principle. However, recent developments have indicated a diversification of this approach, including the application of HIIT, interventions that take advantage of the local environment, mobile app- and home-based rehabilitation models, and comprehensive programs that include psychosocial factors.

The demonstrated clinical effectiveness of HIIT, the benefits of comprehensive psychosocial interventions, and the practical advantages of mobile-based rehabilitation suggest future directions for the scalability and qualitative improvement of Korean CR programs. These results can be used to establish a multidisciplinary, patient-tailored CR model.

## Funding

Not applicable.

## Conflict of Interest

The authors declare that they have no conflicts of interest.
